# Spectrum of AGT (M235T) rs699 and AGTR1 (A1166C) rs5186 gene variants and its association with coronary artery disease in Pakistani patients

**DOI:** 10.12669/pjms.41.4.9993

**Published:** 2025-04

**Authors:** Syed Tousif Ahmed, Muhammad Israr Nasir, Kanwal Fatima Amir, Pirzada Qasim Raza Siddiqui

**Affiliations:** 1 Syed Tousif Ahmed, MBBS, M.Phil Professor & Head, Department of Physiology, Ziauddin University, Karachi, Pakistan; 2 Muhammad Israr Nasir, M.Sc. PhD. Associate Prof Molecular Pathology, Department of Molecular Pathology, Fizaiya Ruth Pfau Medical College Karachi Campus, Air University Islamabad, Pakistan; 3 Kanwal Fatima Amir, MBBS, FCPS Associate Professor, National Institute of Cardiovascular Diseases, Karachi, Pakistan; 4 Pirzada Qasim Raza Siddiqui, M.Sc, M.Phil, PhD Professor Emeritus, Department of Physiology, Ziauddin University, Karachi, Pakistan

**Keywords:** AGT and AGTR1 gene polymorphisms, Coronary artery disease

## Abstract

**Background and Objective:**

Coronary artery disease (CAD) is a multifaceted ailment influenced by genetic and acquired factors. In this study we tried to determine the association of CAD with polymorphisms in renin-angiotensin-aldosterone system (RAAS) genes AGT(M235T) rs699 and AGTRI(A1166C) rs5186.

**Method:**

This case-control study was conducted at Ziauddin University and National Institute of Cardiovascular Diseases Karachi from January, 2019 to June, 2020. It included 239 participants between 30-70years from both genders via convenient sampling. The participants were divided into two groups of 160 controls and 79 angiographically diagnosed CAD patients. Genotyping of AGT(M235T) and AGTRI(A1166C) was investigated by the allele-specific polymerase chain reaction (AS-PCR). Statistical analysis was done using SPSS Version-22. Independent sample t-test was applied for comparison of quantitative variables. The AGT(M235T) and AGRT1(A1166) genes were compared by Chi- square test.

**Results:**

There was no significant association found between CAD and AGT(M235T) gene variants CC, CT and TT (p=0.3; p=0.1; p=0.6 respectively). AGTRI(A1166) of AA and CC variety showed significant association with CAD(p<0.001), while its AC variant showed no significant association with the disease. The odds of CC of AGRT1(A1166C) having CAD were 14 times more, whereas having CAD with AA of AGRT1(A1166C) were 70% less.

**Conclusion:**

Individuals with CC polymorphisms of AGTRI(A1166) gene are 14 times more likely to develop CAD, whereas those with AA variation are less likely to develop the disease. AC variation of the AGTRI(A1166) gene along with all variations of the AGT(M235T) gene were not associated with development of CAD.

## INTRODUCTION

Coronary artery disease (CAD), a major health burden, has numerous gene polymorphisms that can trigger coronary atherosclerosis.^1^ It is expected that by the end of 2025, CAD will be a major source of mortality globally.^2^ As stated by the American Heart and Stroke Statistics Association, in 2022, young people in the USA developed some type of cardiac disease.^3^ One study reported that one in four middle-aged-urban Pakistanis was prone to CAD, in which women above the age of 40 are more prone to the disease.^4^ It is generally due to modifiable risk factors including smoking, alcohol ingestion, dysregulated serum lipids, hypertension, obesity, diabetes mellitus, sedentary life style, family history and psychological problems. Moreover, reasons like gender, age, genetics and family history are the foremost consistent risk factors.^5^ The commencement and severity of CAD can basically be determined by the collaboration of various genetic and ecological issues. The renin-angiotensin-aldosterone system (RAAS) controls the systemic blood-pressure The AGT gene is responsible for production of angiotensinogen which is then converted to angiotensin 1 by renin. Hence, this gene produces the substrate which starts the RAAS cascade. It is thus involved in CAD by alterations in systemic blood pressure.^6^,^7^

Another gene responsible for regulating RAAS is the AGTR1 gene which produces angiotensin II Type-1 receptor (AT1R). AT1R mediates the activity of angiotensin II. The production of this critical receptor is regulated by AGTR1 methylation in which adenine is replaced by cytosine at the 1166 position causing hypermethylation, a condition linked to CAD. SNP *rs5186*, an SNP known as +1166A/C or A1166C, is located in the 3’ untranslated region of the angiotensin II receptor Type-1 gene AGTR1. Similarly, in AGT gene methionine is often replaced by threonine at the 235-position, causing overproduction of angiotensinogen. *SNP rs699, in the angiotensin AGT gene* is due to T to C substitution in the exon 2, resulting in a functional methionine (M) to threonine (T) exchange at codon 268 (M268T).^8^ This concept brings us to our study’s primary goal of determining whether an association exists between polymorphisms of these genes and CAD.

METHODS

This was a case-control study. Sample size was calculated to be 239 via OpenEpi calculator and non-probability convenient sampling technique was used.

### Ethical Approval:

It was approved from both institutes (ERC Ref. No.: 07/2019, dated February 16, 2019) from the NICVD and Ziauddin University’s Ethical Review Committee No. 004511TA).

Participants were recruited as 160 controls and 79 CAD patients, aged 35-70years, who were hospitalized to the National Institute of Cardiovascular Disease (NICVD), Karachi between October 2019 to December 2020. These comprised of individuals with acute congestive heart failure, previous heart attack and unstable angina. The CAD-cases were identified via angiography as more than 50% occlusion in at least one of the three vessels. The also assessed for diabetes on basis of fasting blood sugar (FBS) >126mg/dl (Normal-Prediabetic=70-126mg/dl) and random blood sugar (RBS) (Normal<180mg/dl), and BMI (Normal=27.5kg/m^2^) as well as HbA1c (>6.5).^9^,^10^ Healthy volunteers who were selected from the same hospitals as the control group underwent routine examinations and had no prior history of CAD. Individuals with valvular heart disease, cardiomyopathy, cardiac hypertrophy, myocarditis, endocarditis, severe aortic stenosis and those not giving consent were excluded from the study. To verify each subject’s health condition, blood pressure and a resting electrocardiogram (ECG) were also taken. 10ml blood was extracted for biochemical analysis and PCR-based polymorphism of renin-angiotensin-aldosterone system (RAAS) (including AGT (M235T) and AGTRI (A1166C)) was assessed.

### Genetic analysis:

Following the company’s guidelines, the DNA from the distal blood vessels of limbs was extracted from each group utilize Diazole BD Solution (Invitrogen TM). Qubit 2.0 was used to verify the quality and quantity of extracted DNA. DNA was further diluted @ 25ng/ul and 4ul per PCR reaction was used which makes the total to 100ng (InvitrogenTM, QubitTM dsDNA Assay KIT Catalogue No.: 32853). To analyze bAGT (M235T) SNP ID: rs699 and AGTR1 gene polymorphisms SNP ID 5186, a 25μl PCR was performed using Accu Prime™ Taq deoxyribose nucleic acid polymerase (DNA zol^TM^ BD Reagent (Invitrogen) Catalogue No.: 10974020), hundred ng DNA, 1×PCR buffer, 1.5 mM MgCl2, 5 mM dNTP mix, and 10μM Primer in every allele specific polymerase chain reaction (AS-PCR). The PCR reaction conditions for AGT were 94ºC1 min at, followed by one minute at 53ºC, and one minute at 72ºC, 35 cycles. And final extension at 72ºC for 10 min. The PCR reaction was performed in a SCILOGEX TC 1000-G Thermal Cycler. AGTR1 PCR-Cycling was carried out in 35 cycles of amplification, with the first denaturation happening at 94ºC for five minutes, followed by 30 seconds at 94ºC, 30 seconds at 53.2ºC, and 60 seconds at 72ºC.

The genotyping of amplification products of PCR was observed, using ethidium bromide stained 2% agarose gel with 1X TBE and for electrophoresis, 120V for 30 minutes were provided, followed by gel documentation on the Gel Doc. Table-I: Provides an overview of the primer sequences for the two variants. Proforma was used to collect the data. The analysis of statistics was done out using IBM SPSS version 22.0 software. The numeric details were presented as mean ±SD standard deviation whereas frequency and percentages were used to represent categorical data. Shapiro-Wilk test was done for normality of data. As all quantitative variables were normally distributed, independent samples t-test was applied. The Chi square test was used to compare AGT and AGRT1 between the groups, and an odds ratio was computed. The P- value <0.05 and OR >1 was deemed to be statistically important in all analyses. In AGT gene, the C-allele (wild-type) contains 266-basepair and the T allele has 303-basepairs. AGTR1 genotype has 448 bp amplicon size in all variants i.e. AA, AC and CC (wild-type).^11^,^12^

**Table-I:** The primer sequence of two variants with product size.

Gene	SNP	Forward (5′-3′)	Reverse (5′-3′)	Product size(bp)
AGTR 1	A1166 C rs5186	F1:TTCACTACCAAATGAGCA F2:TTCACTACCAAATGAGCC 10pmole/ul (ABAJY, M.Y.,2016)(9)	R:CCTCCACCCTGTTCAGCC 10pmole/ul	F1+R=448bp”A ” F2+R=448bp”C ”
AGT	M235T rs699	F1,GGAAGACTGGCTGCTCCCTTA C F2,GTCCTCTCCCCAACGGCTGTCT 10pmole/ul (Khatami, M.,2017)(10)	R2,AACCTGACCCTTCTGAGTGTA G R1,GTGCTGTCCACACTGGCTCAC A 10pmole/ul	The T alleleF1+R2 490-bp and C allele F2+R1 428-bp fragment

## RESULTS

The study comprised of 239 participants, including 79 coronary artery disease patients and 160 healthy controls. There were 71% males and 29% females in the control group. The cases comprised of 63% males and 37% females. The gender difference between the two groups was not statistically significant (p-value 0.868). A significant association was seen between the two groups on basis of ethnicity (p-value = <0.001) and smoking (p-value = 0.027). No significant difference in systolic or diastolic was observed between the two groups (p-value = 0.051 and 0,105 respectively). The study found a significant difference (p<0.0001) between the family histories of CAD between the two groups (Table-II).

**Table-II:** Sociodemographic attributes of the Instances and Reference Groups.

Demographic attributes		Cases (n=79)		Controls (n=160)		P value
		Mean±SD/ Frequency (%)		Mean±SD/ Frequency (%)		
Age		49.6 ± 9		48 ± 9		0.52**
Gender	Male	56	71%	100	63%	0.868*
	Female	23	29%	60	37%	
Ethnicity	Punjabi	10	13%	10	6%	<0.001*
	Pashtuns	11	13.5%	8	5%	
	Sindhi	9	11.5%	33	21%	
	Urdu Speaking	49	62%	109	68%	
Smoking Status	Yes	23	29%	26	16%	0.027*
	No	56	71%	134	84%
Blood pressure	Systolic	125.9±17.9		121.09 ± 6.9		0.051**
	Diastolic	77.4±10.4		78.5±4.3		0.105
Family History of CVD	Yes	78	99%	30	19%	<0.001*
	No	1	1%	130	81%	

* Chi-square applied,

** Independent sample t-test applied.

The Odds of developing CAD were 14 times more with the variant (CC) of AGRT1 (CI at 95% 6.2-31.4, P value=0.0001). The Odds of developing CAD were 70 percent less with the Gene Polymorphism (AA) of AGRT1 (CI at 95% 0.2- 0.6, P-value=0.0001) (Table-III).

**Table-III:** Assessment of AGT, and AGRT1 gene polymorphism as a Predisposing risk for coronary artery disease.

			CasesControls		Odds Ratio	95% Confidence Interval	P -Value
			n=79	n=160			
Gene AGT M235T rs699	CC	+	22	54	0.8	0.4-1.3	0.3
		-	57	106			
	CT	+	53	92	1.5	0.8-2.6	0.1
		-	26	68			
	TT	+	4	6	1.4	0.3-5.0	0.6
		-	75	154			
Gene AGRT1 A1166C rs5186	AA	+	33	109	0.3	0.2-0.6	0.0001
		-	46	51			
	AC	+	10	30	0.6	0.3-1.4	0.2
		-	69	130			
	CC	+	36	9	14	6.2-31.4	0.0001
		-	43	151			

Chi-square test was applied.

### Genotype distribution of AGT and AGTR1 gene:

The RAAS gene polymorphism assessment reveals the frequency of the angiotensinogen gene (AGT gene) in cases and controls, with the homozygous allele frequency being 28% in cases and 34% in controls. The frequency of heterozygous alleles in cases was 67% in cases, and 57% controls. Whereas in the gene (AGTR1) homozygous (AA) Allele is 33% in cases and 68% in Controls, the homozygous (CC) allele is 10% in cases and 68% in controls. The frequency of the heterozygous (AC) allele is 12% in cases and 19% in controls.

## DISCUSSION

Currently, researchers are investigating the relationship between RAAS gene and CAD, an entity in which arteries constrict or clog due to the intricate mechanism.^12^ Our study highlights this link by focusing on RAAS gene polymorphisms. The study used AS-PCR to assess RAAS gene variations such as AGT (M235T), and AGTRI (116C). We found no significant association of these polymorphisms with gender. This finding is in coherence with other studies.^13^,^14^ We also report Urdu speakers as the ethnic population at highest risk in developing CAD following by Punjabis; a finding similar to Ashraf et al, 2017.^15^

We also found that smokers were more likely to be involved in cardiovascular events, consistent with previous research and the risk of stable CAD.^16^ Family history was significantly associated with development of CAD. Similar findings were also observed in previous studies.^17^ The mean BMI values show no significant difference between cases and controls(p=0.051). Contradictory to our findings, studies have shown that there is a strong link between obesity and the risk of CAD.^18^ In our study community, the systolic blood pressure between the groups was not significantly different between the groups. Other researchers found that cardiovascular risks is directly proportional to systolic and diastolic BP.^19^ Patients with low ejection fraction (EF) have higher mortality and more frequent major cardiac events as compared to those with EF=66% or above as reported by Liu et al, 2022.^20^ The study reported the CC genotype in A1166C polymorphism of the AGTR1 gene variability as an inducer of endothelium associated abnormalities in structural and functional heart condition among individuals who had arterial hypertension.^21^

In current study homozygous CC allele (46%) was more frequent in cases whereas in controls AA (68%) was more common. Other studies have reported that patients with higher C allele and CC genotype frequencies are linked to greater risk of CAD development.^22^ The association between obesity and AGTR1 (A1166C) gene was not established between obesity and control groups, the frequency of the CC genotype and C allele, but not the AA genotype and T allele. A meta-analysis found that AT1R homozygotes of the C allele showed higher TID values, while AT1R heterozygotes showed higher LHR, suggesting the A1166C polymorphism isn’t necessarily linked to HF susceptibility.^23^

Our results suggest that the odds of developing CAD are 14 times higher for the (CC) genotype, whereas the (AA) genotype had 30% less probability. The Iranian population has been found to be significantly affected by both the (CC) and (AA) genotypes, indicating a complex genetic profile.^24^ This is in contrast to our results. In a current study we found frequent heterozygous CT allele (67%) in cases, while frequent CT allele (57%) in controls. A study in Turkey, found that the AGT rs699 TT genotype significantly correlated in PAD patients.^25^ Research in Egypt reveals, AGT and ACE genetic variants similar to our study, influence CAD hazard incidence, either separately or in combination.^26^ M235T (rs699) variants, characterized by a thymine to cytosine substitution at base 704, are linked to higher serum AGT contents and elevated pressure in individuals with the TT allele.^27^ Genetic variants, including Insertion-deletion and M235T mutations, interact with other genetic variants and risk factors, causing ethnic and regional inequalities in disease-specific polymorphisms.

### Limitations

There were some limitations such as smaller number of patients, financial and time-consuming involvement, but it is the need researcher should have focused on specific new genomic sites in our population. Quantification of variant load was also not seen due to budget limitations.

## CONCLUSION

Individuals with CC polymorphisms of AGTRI(A1166) gene are 14 times more likely to develop CAD, whereas those with AA variation are less likely to develop the disease. AC variation of the AGTRI(A1166) gene along with all variations of the AGT(M235T) gene were not associated with development of CAD.

**Fig.1:**
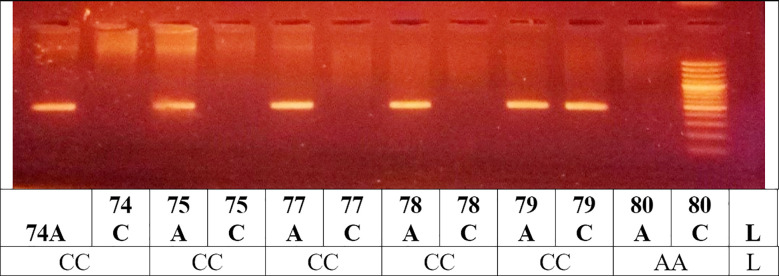
Lane (L) indicate 100bp DNA: AA 448 bp, CC 448 bp, AC 448 bp AGTR1 Gene Polymorphism Genotyping (alleles were identified by the presence of homozygous AA 448 bp or CC 448 bp fragments, respectively. Heterozygous AC genotype shows the presence of bands at 448)

**Fig.2:**
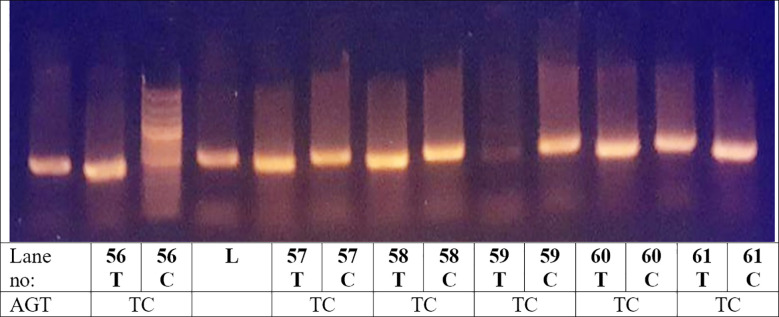
Lane (L) indicate 100bp DNA: The C allele appeared as a 428-bp fragment and the mutated T allele as a 490 bp. fragment. AGT Gene Polymorphism Genotyping. The C allele was represented by a 428-bp fragment, whereas the mutant T allele was represented by a 490-bp fragment. UV trans illuminator was used to record the products of PCR.

### Author Contributions:

**STA:** Designed and conducted the research, gathered, validated and analyzed the data, and developed the initial manuscript.

**MIN:** Designed the work, analyzed the data and closely supervised and monitored all aspects of this study from conception of the idea paper submission.

**KFA:** Selected and sorted out the patients, obtained samples and critically analyzed the manuscript.

**PQRS:** supervised the research, and critically revised and edited the manuscript.
